# Unraveling the Complex Interplay of Fis and IHF Through Synthetic Promoter Engineering

**DOI:** 10.3389/fbioe.2020.00510

**Published:** 2020-06-18

**Authors:** Lummy Maria Oliveira Monteiro, Ananda Sanches-Medeiros, Cauã Antunes Westmann, Rafael Silva-Rocha

**Affiliations:** Ribeirão Preto Medical School (FMRP), University of São Paulo, Ribeirão Preto, Brazil

**Keywords:** regulatory network, *cis*-regulatory elements, complex promoters, global regulators, transcriptional crosstalk, fine-tuning

## Abstract

Bacterial promoters are usually formed by multiple *cis*-regulatory elements recognized by a plethora of transcriptional factors (TFs). From those, global regulators are key elements since these TFs are responsible for the regulation of hundreds of genes in the bacterial genome. For instance, Fis and IHF are global regulators that play a major role in gene expression control in *Escherichia coli*, and usually, multiple *cis*-regulatory elements for these proteins are present at target promoters. Here, we investigated the relationship between the architecture of the *cis*-regulatory elements for Fis and IHF in *E. coli*. For this, we analyze 42 synthetic promoter variants harboring consensus *cis*-elements for Fis and IHF at different distances from the core −35/−10 region and in various numbers and combinations. We first demonstrated that although Fis preferentially recognizes its consensus *cis*-element, it can also recognize, to some extent, the consensus-binding site for IHF, and the same was true for IHF, which was also able to recognize Fis binding sites. However, changing the arrangement of the *cis*-elements (i.e., the position or number of sites) can completely abolish the non-specific binding of both TFs. More remarkably, we demonstrated that combining *cis*-elements for both TFs could result in Fis and IHF repressed or activated promoters depending on the final architecture of the promoters in an unpredictable way. Taken together, the data presented here demonstrate how small changes in the architecture of bacterial promoters could result in drastic changes in the final regulatory logic of the system, with important implications for the understanding of natural complex promoters in bacteria and their engineering for novel applications.

## Introduction

Bacteria have evolved complex gene regulatory networks to coordinate the expression level of each gene in response to changing environmental conditions. In this aspect, a typical bacterium such as *Escherichia coli* uses around 300 different transcriptional factors (TFs) to control the expression of more than 5,000 genes, and gene regulation in bacteria has been extensively investigated in the last six decades (Lozada-Chavez, 2006). Among the known TFs from *E. coli*, global regulators are able to control the highest percentage of transcriptional units in response to significant physiological or environmental signals, such as the metabolic state of the cell, the availability of carbon sources, and the presence of oxygen (Martínez-Antonio et al., 2003; Ishihama, 2010), while local regulators are responsible for gene regulation in response to specific signals (such as sugars and metals) (Ishihama, 2010; Browning and Busby, 2016). Most TFs control gene expression through their interaction with specific DNA sequences located near the promoter region, the *cis*-regulatory element, or transcriptional factor binding site (Browning and Busby, 2004, 2016). Over the decades, many *cis*-regulatory elements for many TFs from *E. coli* have been experimentally characterized, mapped, and compiled in databases such as RegulonDB and EcoCyc (Gama-Castro et al., 2016; Keseler et al., 2017). Analysis of these datasets demonstrates that TFs usually act in a combinatorial way to control gene expression, where multiple *cis*-regulatory elements for different TFs are located in the upstream region of the target genes (Guazzaroni and Silva-Rocha, 2014; Rydenfelt et al., 2014; Gama-Castro et al., 2016). Therefore, the arrangement of *cis*-regulatory elements at the target promoters is crucial to determine which TFs will be able to control the target gene and how these regulators interact with each other once bound to the DNA (Collado-Vides et al., 1991; Ishihama, 2010).

Several studies have explored the relationship between the architecture of *cis*-regulatory elements and the final logic of the target promoters, and initial attempts have focused on the mutation of *cis*-regulatory elements from natural promoters to investigate how these elements specify the promoter activity dynamics (Sawers, 1993; Darwin and Stewart, 1995; Izu et al., 2002; Setty et al., 2003). More recently, synthetic biology approaches have been used to construct artificial promoters through the combination of several *cis*-regulatory elements, and these have been characterized to decipher their architecture/dynamics relationship (Cox et al., 2007; Isalan et al., 2008; Kinkhabwala and Guet, 2008; Shis et al., 2014). However, while most synthetic biology approaches have focused on *cis*-elements for local regulators (which do not commonly regulate gene expression in a combinatorial manner), we recently investigated this combinatorial regulation problem with global regulators (Guazzaroni and Silva-Rocha, 2014; Amores et al., 2015; Monteiro et al., 2018). This is important because global regulators (such as IHF, Fis, and CRP) have numerous binding sites along the *E. coli* genome and frequently co-occur at target promoters (Guazzaroni and Silva-Rocha, 2014). Thus, Fis and IHF are two global regulators that play a critical role in coordinating gene expression in *E. coli* as well as in mediating DNA condensation in the cell (Azam and Ishihama, 1999; Browning and Busby, 2004; Browning et al., 2010; Ishihama, 2010). Fis, an abundant nucleoid-associated protein (NAP), is related to gene expression regulation in fast-growing cells, varying its function (as a repressor or activator transcriptional factor) according to its biding site position related to the core promoter (Hirvonen et al., 2001), while IHF is a NAP, which activity relates to changes in gene expression in cells during the transition from exponential to stationary phase (Azam and Ishihama, 1999; Azam et al., 2000; Browning et al., 2010). Moreover, IHF binds to AT-rich DNA motifs with well-defined sequence preferences, while Fis also prefers AT-rich regions with a more degenerate sequence preference (Déthiollaz et al., 1996; Ussery et al., 2001; Dorman and Deighan, 2003; Aeling et al., 2006). Additionally, cross-regulation between Fis and IHF has been demonstrated for several systems, and how specific vs. promiscuous DNA recognition can be achieved for these two global regulators is not fully understood (Browning et al., 2010; Ishihama, 2010; Rossiter et al., 2015).

We previously explored how complex synthetic promoters harboring *cis*-regulatory elements for CRP and IHF can generate diverse regulatory logic depending on the final architecture of synthetic promoters, demonstrating that it is not possible to predict the regulatory logic of complex multiple promoters from the known dynamics of their simple versions (Monteiro et al., 2018). Here, we further explore this approach to investigate the relationship between *cis*-regulatory elements for Fis and IHF. Using consensus binding sites for these 2 TFs at different promoter positions and in different numbers, we first demonstrated that while some promiscuous interactions occur between the TFs and the binding sites, some specific *cis*-regulatory architectures can completely abolish non-specific interactions. Additionally, complex promoters constructed by the combination of *cis*-elements for Fis and IHF can generate many completely different outputs, such as Fis-repressed promoters, IHF-repressed promoters, and systems where Fis and IHF act as activators. As these changes in promoter logic result from changes in promoter architecture only (and not on the affinity of the transcriptional factor to each individual *cis-element*), the data presented here reinforce the notion that complex bacterial promoters can display emergent properties, where their final behavior cannot be defined from the characterization of the individual component. Taken together, our findings present a comprehensive strategy for fine-tuning gene circuits to perform optimally in a given context (e.g., engineering of synthetic promoters) as well as provide insights for the understanding of natural complex promoters controlled by global regulators.

## Results and Discussion

### Generation of Complex Promoters for Fis and IHF

**Figure 1:**
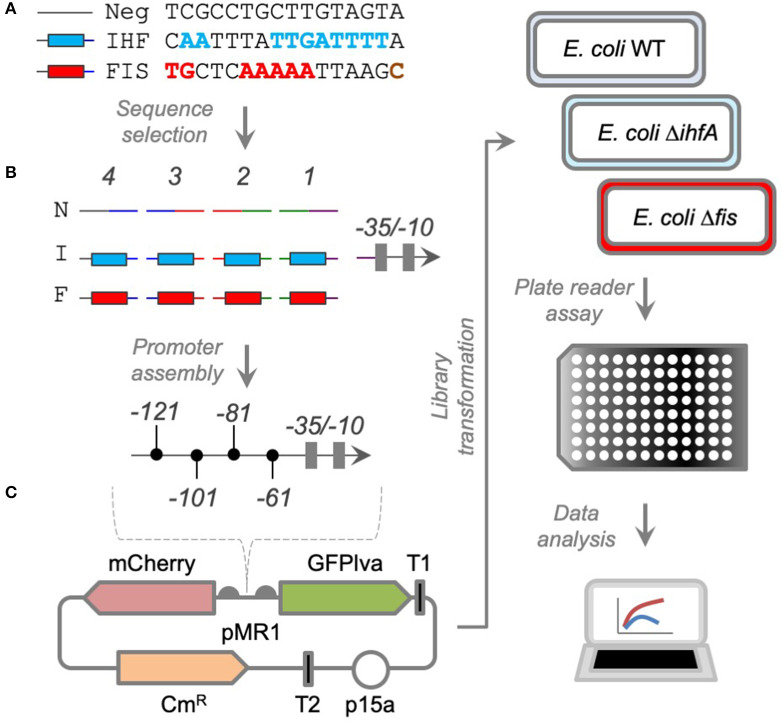
Strategies to construct synthetic complex promoters. **(A)** DNA sequences harboring the consensus sequence for IHF or Fis binding were selected, along with a control sequence that cannot be recognized by any TF. **(B)** Double-stranded DNA fragments were produced with cohesive ends specific for each promoter position (numbered from 1 to 4) and assembled together with a weak core promoter harboring the −35/−10 boxes for RNAP recognition (Guazzaroni and Silva-Rocha, 2014). **(C)** The fragments were cloned into a promoter probe vector (pMR1) harboring resistance to chloramphenicol (CmR), a medium-copy number origin of replication (p15a), and two reporter genes (mCherry and GFPlva). The libraries were introduced into wild-type and mutant strains of E. coli from the KEIO collection (Baba et al., 2006). The resultant strains were analyzed at the population level in a plate reader and the data processed using script in R.

In order to investigate the effect of promoter architecture in the regulation by Fis and IHF, we evaluated the effect of 12 complex promoters constructed in early work (Monteiro et al., 2018) and we constructed 30 new combinatorial promoters with consensus DNA sequences for Fis (Fis-BS) and IHF (IHF-BS) binding sites positioned upstream of a weak core promoter (−35/−10 region) at specific positions (1–4) centered at the −61, −81, −101, and −121 regions related to the transcriptional start site (TSS) (Figure 1). For that, we generated double-strand DNA sequences for Fis-BS, IHF-BS, and a neutral sequence (Neg) with no related transcriptional binding site, which were combined for the generation of a library of synthetic promoters, merging the transcriptional binding sites for Fis, IHF, and/or neutral sequence for each position (Table 1). The complex promoters were assembled by DNA ligation and cloned into pMR1, a mid-copy number vector harboring mCherry and GFPlva as reporter fluorescent proteins (Figure 1). The resulting reporter plasmids (with each promoter controlling only by GFPlva expression) were used to transform competent *E. coli* wild-type strain (BW25113—WT) and/or *E. coli* mutants for *ihfA* (Δ*ihfA*) and *fis* (Δ*fis*) (from Keio collection) (Baba et al., 2006). Using these constructs, we assayed promoter activity for 8 h in minimal media (M9 complete), measuring the relative GFP expression (GFP/OD) in all strains in the plate reader fluorimeter Victor X3 (PerkinElmer). As a negative control, we used the Neg sequence occupying the 4 possible positions before the core promoter. All data presented in this work are referred to 4 h of cell growth. In the next sections, we present the results of the promoter analysis per category to uncover the *cis*-regulatory logic for each variant.

**Table 1:** Strains, plasmids, and primers used in this study.

**Strains, plasmids, and primers**	**Description**	**References**
**Strains**		
*E. coli* DH10B	*F^−^ endA1 deoR^+^ recA1 galE15 galK16 nupG rpsL Δ(lac)X74 ϕ80lacZΔM15 araD139 Δ(ara,leu)7697 mcrA Δ(mrr-hsdRMS-mcrBC) Str^*R*^ λ^−^*	Sambrook et al., 1989
*E. coli* BW25113	*lacI+rrnBT14 ΔlacZWJ16 hsdR514 ΔaraBADAH33 ΔrhaBADLD78 rph-1 Δ(araB–D)567 Δ(rhaD–B)568 ΔlacZ4787(::rrnB-3) hsdR514 rph-1*	Datsenko and Wanner, 2000
*E. coli* JW1702	*E. coli* BW25113 with *ΔihfA* mutation	Baba et al., 2006
*E. coli* JW3229	*E. coli* BW25113 with *Δfis* mutation	Baba et al., 2006
**Plasmids**		
pMR1	Cm^R^; orip15a; Promoter probe vector with mCherry and GFPlva reporters	Guazzaroni and Silva-Rocha, 2014
pMR1-NNNN	pMR1 with a reference promoter with 4 non-regulatory sequences	Monteiro et al., 2018
pMR1-FNNN	pMR1 with a synthetic promoter with a Fis *cis*-elements at position 4	This study
pMR1-NFNN	pMR1 with a synthetic promoter with a Fis *cis*-elements at position 3	This study
pMR1-NNFN	pMR1 with a synthetic promoter with a Fis *cis*-elements at position 2	This study
pMR1-NNNF	pMR1 with a synthetic promoter with a Fis *cis*-elements at position 1	This study
pMR1-NNFF	pMR1 with a synthetic promoter with Fis *cis*-elements at positions 2 and 1	This study
pMR1-FNNF	pMR1 with a synthetic promoter with Fis *cis*-elements at positions 4 and 1	This study
pMR1-FFNN	pMR1 with a synthetic promoter with Fis *cis*-elements at positions 4 and 3	This study
pMR1-NFFN	pMR1 with a synthetic promoter with Fis *cis*-elements at positions 3 and 2	This study
pMR1-NFNF	pMR1 with a synthetic promoter with Fis *cis*-elements at positions 3 and 1	This study
pMR1-FNFN	pMR1 with a synthetic promoter with Fis *cis*-elements at positions 4 and 2	This study
pMR1-FFNF	pMR1 with a synthetic promoter with Fis *cis*-elements at positions 4, 3 and 1	This study
pMR1-FNFF	pMR1 with a synthetic promoter with Fis *cis*-elements at positions 4, 2 and 1	This study
pMR1-NFFF	pMR1 with a synthetic promoter with Fis *cis*-elements at positions 3, 2 and 1	This study
pMR1-FFFN	pMR1 with a synthetic promoter with Fis *cis*-elements at positions 4, 3 and 2	This study
pMR1-FFFF	pMR1 with a synthetic promoter with Fis *cis*-elements at positions 4, 3, 2 and 1	This study
pMR1-INNN	pMR1 with a synthetic promoter with a IHF cis-element at position 4	Monteiro et al., 2018
pMR1-NINN	pMR1 with a synthetic promoter with a IHF cis-element at position 3	Monteiro et al., 2018
pMR1-NNIN	pMR1 with a synthetic promoter with a IHF cis-element at position 2	Monteiro et al., 2018
pMR1-NNNI	pMR1 with a synthetic promoter with a IHF cis-element at position 1	Monteiro et al., 2018
pMR1-IINN	pMR1 with a synthetic promoter with IHF cis-elements at positions 4 and 3	Monteiro et al., 2018
pMR1-NIIN	pMR1 with a synthetic promoter with IHF cis-elements at positions 3 and 2	Monteiro et al., 2018
pMR1-NNII	pMR1 with a synthetic promoter with IHF cis-elements at positions 2 and 1	Monteiro et al., 2018
pMR1-ININ	pMR1 with a synthetic promoter with IHF cis-elements at positions 4 and 2	Monteiro et al., 2018
pMR1-NINI	pMR1 with a synthetic promoter with IHF cis-elements at positions 3 and 1	Monteiro et al., 2018
pMR1-INNI	pMR1 with a synthetic promoter with IHF cis-elements at positions 4 and 1	Monteiro et al., 2018
pMR1-IIII	pMR1 with a synthetic promoter with IHF cis-elements at positions 4, 3, 2 and 1	Monteiro et al., 2018
pMR1-FNNI	pMR1 with a synthetic promoter with a IHF *cis*-element at position 1 and Fis *cis*- element at position 4	This study
pMR1-NFNI	pMR1 with a synthetic promoter with a IHF *cis*-element at position 1 and Fis *cis*- element at position 3	This study
pMR1-NNFI	pMR1 with a synthetic promoter with a IHF *cis*-element at position 1 and Fis *cis*- element at position 2	This study
pMR1-NFFI	pMR1 with a synthetic promoter with a IHF *cis*-element at position 1 and Fis *cis*- elements at positions 3 and 2	This study
pMR1-FFNI	pMR1 with a synthetic promoter with a IHF *cis*-element at position 1 and Fis *cis*- elements at positions 4 and 3	This study
pMR1-IFNN	pMR1 with a synthetic promoter with a IHF *cis*-element at position 4 and Fis *cis*- element at position 3	This study
pMR1-INFN	pMR1 with a synthetic promoter with a IHF *cis*-element at position 4 and Fis *cis*- element at position 2	This study
pMR1-INNF	pMR1 with a synthetic promoter with a IHF *cis*-element at position 4 and Fis *cis*- element at position 1	This study
pMR1-IFFN	pMR1 with a synthetic promoter with a IHF *cis*-element at position 4 and Fis *cis*- elements at positions 3 and 2	This study
pMR1-IFNF	pMR1 with a synthetic promoter with a IHF *cis*-element at position 4 and Fis *cis*- elements at positions 3 and 1	This study
pMR1-INFF	pMR1 with a synthetic promoter with a IHF *cis*-element at position 4 and Fis *cis*- elements at positions 2 and 1	This study
pMR1-IFFF	pMR1 with a synthetic promoter with a IHF *cis*-element at position 4 and Fis *cis*- elements at positions 3, 2 and 1	This study
pMR1-IFFI	pMR1 with a synthetic promoter with a IHF *cis*-elements at positions 4 and 1. Fis *cis*- elements at positions 3 and 2	This study
pMR1-IFNI	pMR1 with a synthetic promoter with a IHF *cis*-elements at positions 4 and 1. Fis *cis*- element at position 3	This study
pMR1-INFI	pMR1 with a synthetic promoter with a IHF *cis*-elements at positions 4 and 1. Fis *cis*- element at position 2	This study
**Primers**		
P1-N5	AATTCTCGCCTGCTTGTAGTA^*^	Monteiro et al., 2018
P1-N3	CGCCTACTACAAGCAGGCGAG	Monteiro et al., 2018
P2-N5	GGCGTCGCCTGCTTGTAGTA	Monteiro et al., 2018
P2-N3	GCGGTACTACAAGCAGGCGA	Monteiro et al., 2018
P3-N5	CCGCTCGCCTGCTTGTAGTA	Monteiro et al., 2018
P3-N3	CCAATACTACAAGCAGGCGA	Monteiro et al., 2018
P4-N5	TTGGTCGCCTGCTTGTAGTA	Monteiro et al., 2018
P4-N3	CAAGTACTACAAGCAGGCGA	Monteiro et al., 2018
P1-I5	AATTCCAATTTATTGATTTTA^*^	Monteiro et al., 2018
P1-I3	CGCCTAAAATCAATAAATTGG	Monteiro et al., 2018
P4-I5	TTGGCAATTTATTGATTTTA	Monteiro et al., 2018
P4-I3	CAAGTAAAATCAATAAATTG	Monteiro et al., 2018
P1-F5	AATTCTGCTCAAAAATTAAGC^*^	This study
P1-F3	CGCCGCTTAATTTTTGAGCAG	This study
P2-F5	GGCGTGCTCAAAAATTAAGC	This study
P2-F3	GCGGGCTTAATTTTTGAGCA	This study
P3-F5	CCGCTGCTCAAAAATTAAGC	This study
P3-F3	CCAAGCTTAATTTTTGAGCA	This study
P4-F5	TTGGTGCTCAAAAATTAAGC	This study
P4-F3	CAAGGCTTAATTTTTGAGCA	This study
CoreP-5	CTTGAGGCACCCCAGGCTTTACACTTTATGCTTCCGGCTCGTATGTTGTGTGGAG	Monteiro et al., 2018
CoreP-3	GATCCTCCACACAACATACGAGCCGGAAGCATAAAGTGTAAAGCCTGGGGTGCCT^*^	Monteiro et al., 2018
pMR1-F	CTCGCCCTTGCTCACC	Monteiro et al., 2018
pMR1-R	ACAAGAATTGGGACAACTCC	Monteiro et al., 2018

* *Restriction sites are underlined in the primer sequences*.

### Changing the Fis Binding Site Architecture Modulates Fis and IHF Binding Specificity

**Figure 2:**
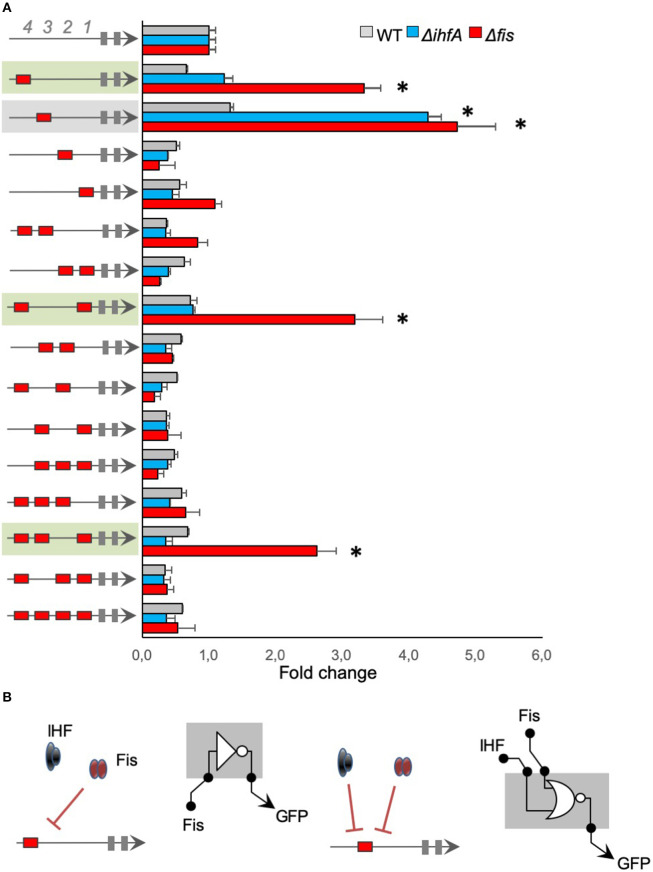
The activity of promoters harboring Fis-binding sites **(A)** The architecture of the synthetic promoter is shown on the left (red boxes represent Fis-BS). Promoter activities are shown in bars and normalized based on the activity of the reference promoter (i.e., a promoter with 4 neutral sequences). Promoter analyses were performed for 4 h of growth, three genetic backgrounds of *E. coli* (wild type—gray bars, Δ*fis—*red bars, and Δ*ihfA—*blue bars). Promoters that displayed a significant increase in activity compared to the wild-type strain were shaded in green or gray for easy viewing. Statistical differences between synthetic promoters and their control (wild type condition) are highlighted by (*) as analyzed using Student's *t*-test with *p* < 0.05. **(B)** Summary of most significant changes in promoter architecture leading to changes in promoter logic.

We analyzed the architecture effect for Fis *cis*-regulatory elements by evaluating the influence of position and sequence combination for Fis-BS. For that, we used promoters merging Fis-BS and Neg sequences to measure relative GFP expression (GFP/OD) levels after 4 h of cell growth in wild-type, Δ*fis*, and Δ*ihfA E. coli* strains, and normalized the results to our negative control (top bars in Figure 2). The results displayed in Figure 2 show that most of the promoters harboring Fis-BS exhibit low activity in wild-type *E. coli*, comparable to the negative control. However, when these promoters were assayed in *E. coli* Δ*fis* strain (red bars), 4 of them displayed a significant increase in activity compared to the wild-type strain (green and gray in Figure 2). Particularly, in the presence of Fis protein, Fis could occupy Fis-BS and act as a repressor of promoter activity. However, not all architectures with Fis-BS at the 4th or 3rd positions display this promoter behavior. This phenomenon only occurs in two other cases with more than 1 Fis-BS combination (promoters shaded in green in Figure 2). This reveals a complex association between promoter architecture and expression profile, which seems to be dependent on the Fis-BS position and arrangement.

We also assayed Fis-BS promoters in the *E. coli* Δ*ihfA* strain (blue bars) to evaluate the specificity of Fis for Fis-BS. Strikingly, despite most promoters display similar activity levels in the Δ*ihfA* strain as in the wild-type, 1 single promoter variant harboring Fis-BS at the 3rd position (−101 relative to the TSS) displayed a substantial increase in activity in the *ihfA* mutant relative to the wild-type strain (promoter shaded in gray in Figure 2). This result indicates that IHF also acts as a repressor of this promoter variant. Although it was restricted to a single promoter variant, these results suggest that non-specific IHF binding to the Fis-BS exists, suggesting that promiscuous regulatory interaction could occur and seems to be dependent on promoter architecture, since this phenomenon is detected only for Fis-BS at the 3rd position. Altogether, these results suggest a complex interplay between the position and combination of Fis-BS and the regulation of gene expression.

### IHF Binding Sites Can Be Recognized by the Fis Regulator in an Architecture-Dependent Manner

**Figure 3:**
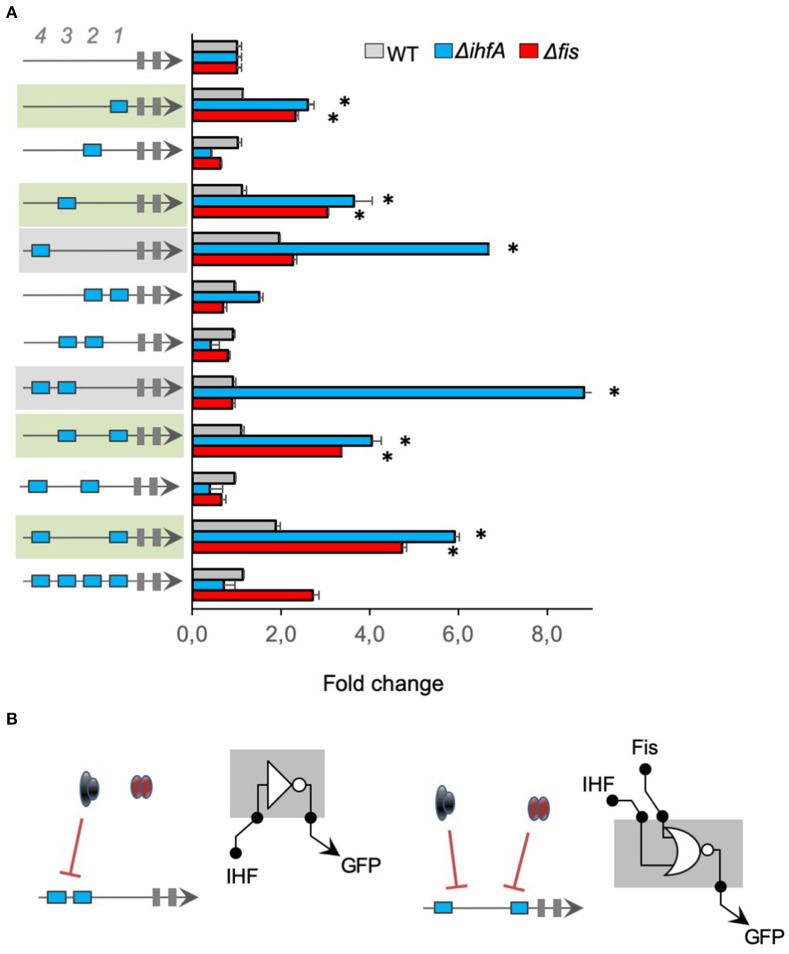
The activity of promoters harboring IHF-binding sites. **(A)** The architecture of the synthetic promoter is shown on the left (blue boxes represent IHF-BS). Promoter activities are shown in bars and normalized based on the activity of the reference promoter (i.e., a promoter with 4 neutral sequences). Eleven promoter variants previously described (Monteiro et al., 2018) were analyzed in wild type (gray bars) Δ*fis* (red bars), and Δ*ihfA* (blue bars) mutant strains of *E. coli*. Promoter architectures that displayed higher activity in *E. coli* Δ*fis* and Δ*ihfA* are shaded in green, indicating that Fis was also able to repress these promoter variants, highlighting a possible crosstalk. Promoter that displayed a strong repression by IHF but no modulation by Fis are shaded in gray, reinforce that the gene expression pattern and the promiscuous or specific binding to transcriptional factors allows for the fine-tuning of promoter activities based on their architectures. Statistical differences between synthetic promoters and their control (wild type condition) are highlighted by (*) as analyzed using Student's *t*-test with *p* < 0.05. **(B)** Summary of most significant changes in promoter architecture leading to changes in promoter logic. Statistical differences between synthetic promoters and their control are highlighted by (*) as analyzed using Student's *t*-test with *p* < 0.05.

Using the same strategy as in Figure 2, we investigated the regulatory logic of promoters harboring multiple *cis-*regulatory elements for IHF, merging IHF-BS, and Neg sequences. Figure 3 shows that most promoters displayed low activity in the wild type strain of *E. coli* and higher activity in *E. coli* Δ*ihfA* (blue bars), in agreement with previous data on complex IHF promoters (green and gray shaded) (Monteiro et al., 2018). However, when these promoters were assayed in *E. coli* Δ*fis* strain (red bars), we observed that 4 promoter architectures also displayed higher activity in this mutant (promoters shaded in green in the figure), indicating that Fis was also able to repress these promoter variants, highlighting a possible crosstalk (Cepeda-Humerez et al., 2015; Friedlander et al., 2016) between these 2 TFs, which should be further investigated in the future. However, it is worth noticing that the promoter variants harboring *cis*-regulatory elements for IHF at 4th or 3rd and 4th positions (−101 and −121 relative to the TSS) displayed both a strong repression by IHF but no modulation by Fis (promoter shaded in gray in Figure 3). Again, these results reinforce that the gene expression pattern and the promiscuous or specific binding to transcriptional factors allows for the fine-tuning of promoter activities based on their architectures.

### Merging IHF-BS and Fis-BS Leads to an Unpredictable Expression Pattern

**Figure 4:**
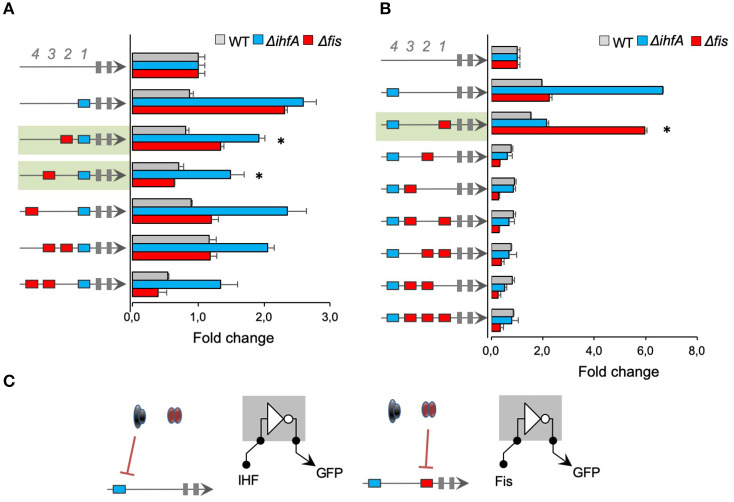
The activity of promoters with combined IHF- and Fis-binding sites. The architecture of the synthetic promoter is shown on the left (blue boxes represent IHF-BS and red boxes represent Fis-BS). Promoter activities are shown in bars and normalized based on the activity of the reference promoter (i.e., a promoter with 4 neutral sequences). Promoter variants were analyzed in wild type (gray bars) Δ*fis* (red bars), and Δ*ihfA* (blue bars) mutant strains of *E. coli*. **(A)** Characterization of promoters with a single IHF-BS fixed at position 1 (−61) and varying Fis-BS. Promoters shaded in green present reduced activity in the *ihfA* and *fis* mutant strains, compared to IHF-BS at the 1st position. Statistical differences between synthetic promoters and their control (PNNNI) are highlighted by (*) as analyzed using Student's *t*-test with *p* < 0.05. **(B)** Characterization of promoters with a single IHF-BS fixed position 4 (−121) and varying Fis-BS. Promoter shaded in green displayed increased activity in the *E. coli* Δ*fis* strain, while it showed no activity in the wild type and Δ*ihfA* strains. Statistical differences between this promoter in Δ*fis* condition and wild type and Δ*ihfA* are highlighted by (*) as analyzed using Student's *t*-test with *p* < 0.05. **(C)** Summary of most significant changes in promoter architecture leading to changes in promoter logic. Statistical differences between synthetic promoters and their control are highlighted by (*) as analyzed using Student's *t*-test with *p* < 0.05.

After we investigated the regulatory interactions for promoters harboring *cis*-regulatory elements for a single transcriptional factor (IHF or Fis), we constructed promoters combining binding sites for both TFs and Neg sequences. In order to systematically investigate the effect of combined transcriptional factor-binding sites on promoter logic, we first fixed 1 IHF-BS at the 1st position (−61) and varied Fis-BS for the 2nd, 3rd, and 4th positions. As shown in Figure 4A, 1 promoter harboring 1 single IHF-BS at the 1st position showed no activity in the wild-type *E. coli* strain but increased activity in the *fis* and *ihfA* mutant strains. However, adding Fis-BS at the 2nd or 3rd position resulted in promoters with reduced activity in the *ihfA* and *fis* mutant strains, compared to IHF-BS at the 1st position (promoters shaded in green in Figure 4A). Comparison of these green shaded promoters to promoters with 1 single Fis-BS at the 2nd or 3rd positions in Figure 2, we cannot observe any patterns between the merging of binding sites for these transcriptional factors, that is, the activity of promoters consisting of both Fis-BS and IHF-BS is not the sum of behaviors from Fis-BS and IHF-BS individually. When 1 single IHF-BS was fixed at the 4th position (−121), the resulting promoter displayed strong activity in Δ*ihfA* strains (Figure 4B). However, when 1 single Fis-BS was added at the 1st position (−61), the resulting promoter displayed increased activity in the *E. coli* Δ*fis* strain, while it showed no activity in the wild type and Δ*ihfA* strains. Therefore, this promoter architecture may be being repressed, especially by Fis regulator (shaded in green). However, for promoters with Fis-BS fixed at the 1st position (Figure 2), we observed a reduction in the promoter activity in the Δ*fis* strain, demonstrating that the presence of IHF in this specific position may influence a positive expression in the absence of Fis. Finally, the addition of 1 single or multiple Fis-BS at different positions completely blocked promoter activity, and this was not relieved in either Δ*fis* or Δ*ihfA* strains, showing that transcriptional factors and binding site sequences of IHF and Fis contribute to promoter complexity. A mutant for *ihfA* and *fis* should be a compelling model to completely understand this promoter logic, but a mutant for both TFs has proven to be difficult to construct. It is important to note that IHF and Fis, which are transcriptional factors, are also NAPs, so the gene expression identified here could be related to possible changes in the DNA geometry (Déthiollaz et al., 1996). Taken together, these results also suggest that Fis and IHF proteins and their binding sites exert complex regulatory patterns, hampering promoter behavior predictions.

### Combination of Fis and IHF Binding Sites Generates Strong Fis and IHF Activated Promoters

**Figure 5:**
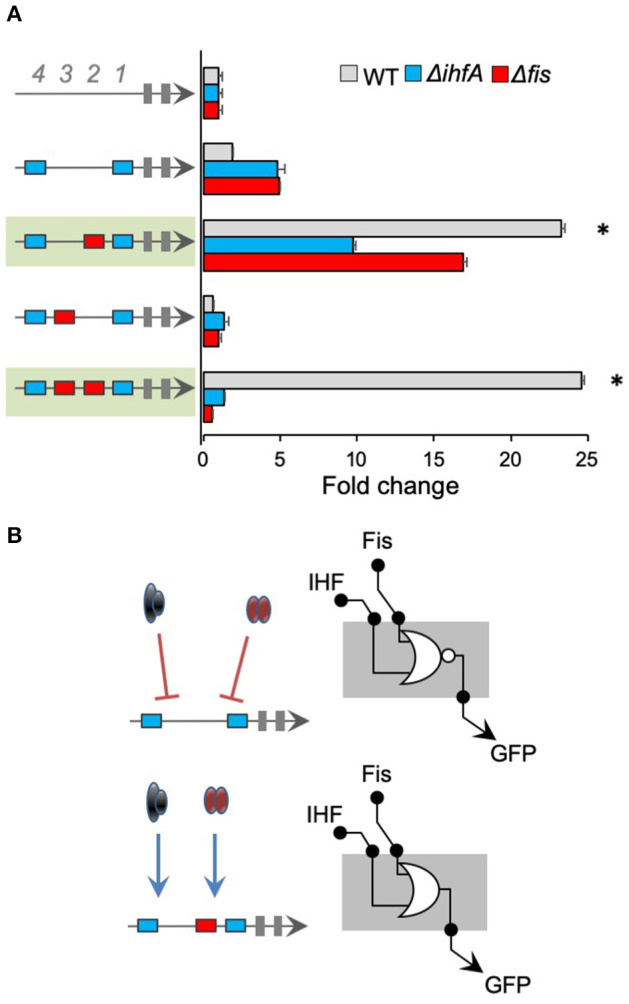
Analysis of promoters with 2 fixed IHF-binding sites. **(A)** The architecture of the synthetic promoter is shown on the left (blue boxes represent IHF-BS and red boxes represent Fis-BS). Promoter activities are shown in bars and normalized based on the activity of the reference promoter (i.e., a promoter with 4 neutral sequences). Promoter variants were analyzed in wild type (gray bars) Δ*fis* (red bars), and Δ*ihfA* (blue bars) mutant strains of *E. coli*. For this analysis, IHF *cis*-regulatory elements were placed at positions 1 and 4, and additional Fis-BS were introduced into the promoters. Promoter that displayed a strong activity in the wild-type when compared to the Δ*fis* or Δ*ihfA* mutant of *E. coli* are shaded in green. Statistical differences between synthetic promoters in wild type condition and Δ*fis* and Δ*ihfA* condition are highlighted by (*) as analyzed using Student's *t*-test with *p* < 0.05. **(B)** Summary of most significant changes in promoter architecture leading to changes in promoter logic. Statistical differences between synthetic promoters and their control are highlighted by (*) as analyzed using Student's *t*-test with *p* < 0.05.

In all promoters presented until this point, while the combination of different *cis*-regulatory genes was able to determine the regulatory logic displayed by IHF and Fis, the 2 TFs acted as repressors of promoter activity (Figures 2–4). However, this behavior shifted when we constructed promoter versions harboring IHF-BS at the 1st and 4th positions and varying sites for Fis-BS (Figure 5). As shown in this figure, when 1 single Fis-BS was added at the 2nd position (−81), the resulting promoter displayed a strong activity in the wild-type strain of *E. coli*, when compared to the version lacking this element (promoter in the green shaded region in Figure 5). Furthermore, when these promoters were assayed in *E. coli* Δ*fis* or Δ*ihfA*, we observed a substantial reduction in their activity, indicating that both TFs acted as activators of the combinatorial promoter. The same behavior was also observed for a promoter harboring 2 IHF-BS (at the 1st and 4th positions) and 2 Fis-BS (2nd and 3rd positions), where reduction in the gene expression was even more evident. The same does not occur for a promoter harboring the 2 sites of IHF-BS and Fis-BS at the 3rd position, indicating the dependence and complexity of the relationship between promoter architecture and gene expression. These results highlight the rise of emergent properties in complex promoters for global regulators (Monteiro et al., 2018), as increasing the number of *cis*-regulatory elements can drastically shift the final regulatory logic of the system.

### Conclusions

**Figure 6:**
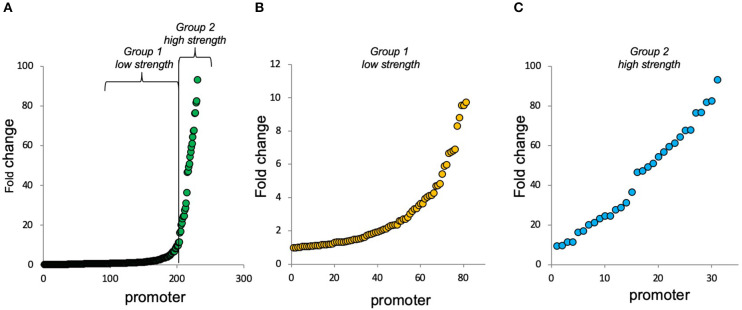
Fold change of complex synthetic promoters at 4 h of growth. Complex promoters' approach could be used as a strategy for the fine-tuning of genetic circuits to perform optimally in a given context. A total of 230 promoter characterizations (74 architectures characterized in different strains and conditions). All promoter details are shown in Table S1. **(A)** All 230 promoter characterizations (from the lowest to the highest fold change) that were divided into 2 groups of interests. **(B)** Promoters with fold change between 1 and 10 (group 1: low strength). **(C)** Promoters with fold change up to 10 (group 2—high strength).

Bacteria are naturally endowed with complex promoters harboring multiple binding sites for several TFs. While several works based on mathematical modeling have argued that combinatorial regulation can be predicted from the characterization of individual promoter elements (Yuh, 1998; Bintu et al., 2005; Hermsen et al., 2006; Zong et al., 2018), along with the previous report (Monteiro et al., 2018) and here we provide growing evidence that small changes in the architecture of *cis*-regulatory elements can drastically change the final response of the system (Kreamer et al., 2016). The unpredictable behaviors observed in these studies might also depict a deeper evolutionary trend in gene regulation that has selected molecular systems/mechanisms capable of promoting both evolvability and robustness of gene expression levels through non-linear gene regulation (Steinacher et al., 2016). Thus, understanding the way the architecture of *cis*-regulatory elements determines gene expression behavior is pivotal not only to understand natural bacterial systems but also to provide novel conceptual frameworks for the construction of synthetic promoters for biotechnological applications (Monteiro et al., 2019b). Frequently, in genetic bioengineering applications, it is also necessary to fine-tune and balance specific gene expression due to the complexity of regulatory networks (Boyle and Silver, 2012; Scalcinati et al., 2012; Steinacher et al., 2016). Several recent studies have focused on the improvement of this strategy for diverse purposes (Egbert and Klavins, 2012; Siegl et al., 2013; Hwang et al., 2018). The present adjusting approach could be used as a strategy for the fine-tuning of genetic circuits to perform optimally in a given context. Our approach provided a library (from this study and from our previous work (Monteiro et al., 2018) of 74 promoter architectures characterized in different strains and conditions for in total of 230 outputs (different promoters in different strains and growth conditions) (Figure 6 and Table S1). Promoters from our synthetic promoter library with small adaptations could be used for diverse purposes in the biotechnological and bacterial network gene regulation fields.

**Figure 7:**
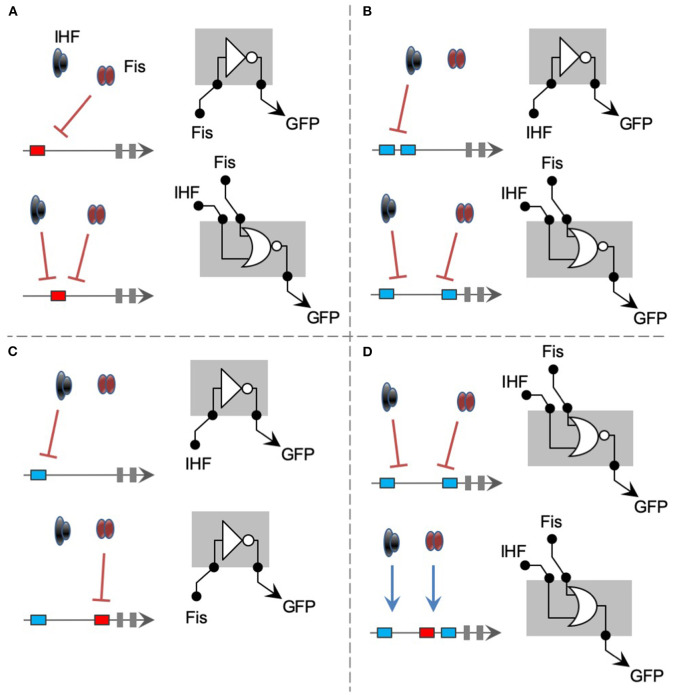
Summary of most significant changes in promoter architecture leading to changes in promoter logic. The figures are represented using logic gate representation for gene regulation, even. **(A)** The change of a single Fis-BS from position −121 to −101 turns a NOT gate for Fis into an NOR gate for Fis and IHF. **(B)** Two IHF-BSs (at positions −121 and −101) worked as a NOT gate for IHF, while changing 1 site to position −61 generates an NOR gate for both IHF and Fis. **(C)** While a single IHF-binding site at position −121 results into a NOT gate for IHF, adding a Fis-binding site to the position −61 creates a NOT gate exclusively dependent on Fis. **(D)** Finally, adding a Fis-BS (position −81) to the NOR gate promoter presented in B drastically changes its logic to an OR gate, where the 2 TFs act as activators.

Abstracting all the gene regulations investigated in this work, we are able to provide a visual summary of the findings reported here from a Boolean logic perspective (Figure 7). As shown in Figure 7A, changing a perfect Fis binding in 20 bp (from position –121 to –101) can turn a specific Fis-repressed promoter into a system repressed by both Fis and IHF. Using a more formal logic gate definition (Amores et al., 2015), this modification can turn a promoter with a NOT logic into one with an NOR logic. However, a promoter harboring 2 IHF-binding sites at positions –121 and –101 displayed specific IHF-repression, while changing the second binding site to position –61 resulted in a promoter repressed by both IHF and Fis (Figure 7B). In terms of promoter logic, this change in cis-element architecture also turns a promoter with NOT logic into one with an NOR logic. When a single IHF-binding site was presented at position –121, the final promoter was only repressed by IHF (Figure 7C). Yet, introducing an additional Fis-binding site at position –61 of this promoter turned it into a system exclusively repressed by Fis. This change maintained the NOT logic of the promoter but changed the TF able to repress the activity. Finally, and more remarkably, while a promoter with 2 IHF-BS (at positions –121 and –61) was repressed by both Fis and IHF, adding a third binding site for Fis at position –81 resulted in a promoter strongly activated by both TFs (Figure 7D). Therefore, this single-change cis-element architecture turned a promoter with NOR logic into an entirely OR promoter responsive to the same TFs. This remarkable regulatory versatility and unpredictability unveiled by synthetic combinatorial promoter shows that we only start to understand the complexity of gene regulation in bacteria. While the work presented here covers two of the main global regulators of *E. coli*, further studies are still necessary to uncover the hidden complexity of combinatorial gene regulation in this bacterium.

## Materials and Methods

### Plasmids, Bacterial Strains, and Growth Conditions

*E. coli* DH10B was used for cloning procedures, while *E. coli* BW25113 was used as the wild-type strain (WT); *E. coli* JW1702-1 was used as a mutant for the IHF transcription factor (TF), and *E. coli* JW3229 was used as a mutant for the Fis TF. All strains were obtained from the Keio collection. For the procedures and analyses, *E. coli* strains were grown in M9 minimal media (6.4 g/L, Na_2_HPO_4_•7H_2_O, 1.5 g/L KH_2_PO_4_, 0.25 g/L NaCl, 0.5 g/L NH_4_Cl) supplemented with chloramphenicol at 34 μg/mL, 2 mM MgSO_4_, 0.1 mM casamino acids, and 1% glycerol as the sole carbon source (Complete M9) at 37°C. Plasmids, bacterial strains, and primers used in this study are listed in Table 1.

### Design of Synthetic Promoter Scaffolds and Ligation Reactions

The construction of synthetic promoters was performed by the ligation reaction of 5′–end phosphorylated oligonucleotides acquired from Sigma Aldrich (Table 1). The design of all single strands was projected to carry a 16 bp sequence containing the Fis binding site (F), IHF binding site (I), or a neutral motif (N), which is a sequence where any TF is able to bind (Figure 1A). These locations were identified as positions 1, 2, 3, and 4, respectively (Figure 1B) and to be located at −61, −81, −101, or −121 bp upstream of the core promoter (Figure 1C). In addition to the 16 bp oligonucleotides, all single strands were designed to contain 3 base pairs overhang for its corrected insertion on the promoter (Figure 1C). Additionally, a core promoter based on the lac promoter, which is a weak promoter and therefore requires activation. The design of the synthetic promoters and the positions of the cis-elements were made based on strategies already performed by our group (Monteiro et al., 2018), aiming to arrange the cis-elements aligned to the transcription initiation site, considering the DNA curvature. To assemble the synthetic promoters, the 5′ and 3′ strands corresponding to each position were mixed at equimolar concentrations and annealed by heating at 95°C for 5 min, followed by gradual cooling to room temperature (25°C) for 5 min, and finally maintained at 0°C for 5 min. The external overhangs of the *cis*-element at position 4 and the core promoter were designed to carry EcoRI and BamHI digested sites. In this way, it was allowed to ligate to a previously digested EcoRI/BamHI pMR1 plasmid. All five fragments (4 *cis*-elements positions plus core promoter) were mixed equally in a pool with the final concentration of 5′ phosphate termini fixed at 15 μM. For the ligase reaction, 1 μL of the fragment pool was added to 50 ng EcoRI*/*BamHI pMR1 digested plasmid in the presence of ligase buffer and ligase enzyme to a final volume of 10 μL. Ligation was performed for 1 h at 16°C, after which the ligase reaction was inactivated for 15 min at 65°C. Two μL of the ligation was used to electroporate 50 μL of *E. coli* DH10B competent cells. After 1-h regenerating in 1 mL LB media, the total volume was plated in LB solid dishes supplemented with chloramphenicol at 34 μg/mL. Clones were confirmed by colony PCR with primers pMR1-F and pMR1-R (Table 1) using pMR1 empty plasmid PCR reaction as further length reference on electrophorese agarose gel. Clones with a potential correct length were submitted to Sanger DNA sequencing to confirm correct promoter assembly.

### Promoter Activity Analysis and Data Processing

Promoter activity was measured for all 42 promoters at different genetic backgrounds and conditions. For each experiment, the plasmid containing the promoter of interest was used to transform *E. coli* wild type, *E. coli* Δ*ihfA* mutant, or *E. coli* Δ*fis* mutant, as indicated. Freshly plated single colonies were selected with sterile loops and then inoculated in 1 mL of M9 media. After 16 h 10 μL of this culture was assayed in 96 wells microplates in biological triplicate with 190 μL of M9 media. Cell growth and GFP fluorescence were quantified using a Victor X3 plate reader (PerkinElmer) that was measured for 8 h at intervals of 30 min. All graphics were constructed based on 4 h of cell growth since under our experimental setup and previous work (Monteiro et al., 2018), most promoters reach maximal activity at 4 h of growth. Therefore, this is the best time point to compare maximal promoter activity. Promoter activities were calculated as arbitrary units dividing the GFP fluorescence levels by the optical density at 600 nm (reported as GFP/OD_600_) after background correction. Technical triplicates and biological triplicates were performed in all experiments. Raw data were processed using *ad hoc* R script (https://www.r-project.org/), and plots were constructed using R (version R-3.6.3). For all analyses, we calculated fold-change expression using pMR1-NNNN as the promoter reference.

## Data Availability Statement

The raw data supporting the conclusions of this article will be made available by the authors, without undue reservation, to any qualified researcher.

## Author Contributions

RS-R and LM designed the experimental strategy. LM, AS-M, and CW performed the experiments. LM, AS-M, CW, and RS-R analyzed and interpreted the data. LM and RS-R wrote the manuscript. All authors have read and approved the final version of the manuscript.

## Conflict of Interest

The authors declare that the research was conducted in the absence of any commercial or financial relationships that could be construed as a potential conflict of interest.
