# Supplementary material for: Unraveling the Complex Interplay of Fis and IHF Through Synthetic Promoter Engineering
Source: Front Bioeng Biotechnol. 2020 Jun 18;8:510. doi: 10.3389/fbioe.2020.00510 (PMC7314903; doi:10.3389/fbioe.2020.00510)
Supplement: Supplementary file 1 [file Table_1.docx]

Unraveling the complex interplay of Fis and IHF through synthetic promoter engineering

Lummy Maria Oliveira Monteiro*, Ananda Sanches-Medeiros, Cauã Antunes Westmann and Rafael Silva-Rocha*

FMRP - University of São Paulo, Ribeirão Preto, SP, Brazil

**Running Title:** Modulating TFBS-Specificity Through Promoter Engineering

**Keywords:** Regulatory network, *cis*-regulatory elements, complex promoters, global regulators, transcriptional crosstalk, fine-tuning

_____________________________________________________________________________

*Correspondence to: Rafael Silva-Rocha, [silvarochar@usp.br](mailto:silvarochar@usp.br)

Lummy Maria Oliveira Monteiro, [lummymaria@gmail.com](mailto:lummymaria@gmail.com)

**Supplementary Table:**

**Table S1.** Complex Synthetic Promoters and their fold change at four hours of growth

| Promoter | Fold  change | Promoter | Fold  change | Promoter | Fold  change | Promoter | Fold  change | Promoter | Fold  change | Promoter | Fold change |
| --- | --- | --- | --- | --- | --- | --- | --- | --- | --- | --- | --- |
| *FNFN* ^c^ | 0.18 | *IFFN* ^b^ | 0.50 | *IFFN* ^a^ | 0.79 | *IIII* ^a^ | 1.14 | *FNNN* ^c^ | 3.33 | *IICC* ^a^ | 59.49 |
| *NFFF* ^c^ | 0.23 | *NNFN* ^a^ | 0.51 | *NIIN* ^c^ | 0.80 | *NFF* ^a^ *I* | 1.17 | *NINI* ^c^ | 3.35 | *IICC* ^b^ | 61.17 |
| *NNFN* ^c^ | 0.25 | *FNFN* ^a^ | 0.52 | *CCNI* ^d^ | 0.81 | *NFFI* ^c^ | 1.18 | *NNNI* ^e^ | 3.55 | *ICCN* ^a^ | 64.39 |
| *IFFN* ^c^ | 0.25 | *FFFF* ^c^ | 0.53 | *NNFI* ^a^ | 0.81 | *NCNI* ^e^ | 1.19 | *NINN* ^b^ | 3.63 | *CNIC* ^b^ | 67.67 |
| *INCI* ^e^ | 0.25 | *FFNI* ^a^ | 0.54 | *ICCN* ^a^ | 0.83 | *FNNI* ^c^ | 1.21 | *INNN* ^a^ | 3.64 | *NCIC* ^a^ | 67.86 |
| *NNFF* ^c^ | 0.27 | *NNCI* ^a^ | 0.54 | *FFNN* ^c^ | 0.83 | *ICNI* ^e^ | 1.22 | *INNI* ^e^ | 3.94 | *NCIC* ^b^ | 76.53 |
| *IFNN* ^c^ | 0.27 | *IFFI* ^c^ | 0.56 | *NNCN* ^a^ | 0.83 | *FNNN* ^b^ | 1.23 | *NINI* ^b^ | 4.05 | *NICC* ^b^ | 76.71 |
| *FNFN* ^b^ | 0.29 | *NNNF* ^a^ | 0.56 | *IFNN* ^b^ | 0.84 | *NFNN* ^a^ | 1.31 | *NCCI* ^e^ | 4.12 | *CINC* ^b^ | 81.80 |
| *IFNF* ^c^ | 0.30 | *NFFN* ^a^ | 0.58 | *ICNI* ^d^ | 0.84 | *CCNI* ^b^ | 1.33 | *NCCI* ^e^ | 4.15 | *NICC* ^a^ | 82.67 |
| *INFN* ^c^ | 0.31 | *FFFN* ^a^ | 0.58 | *ICNI* ^d^ | 0.84 | *NNFI* ^c^ | 1.34 | *NFNN* ^b^ | 4.28 | *CINC* ^a^ | 93.28 |
| *FNFF* ^b^ | 0.32 | *NNCI* ^e^ | 0.59 | *IFFF* ^a^ | 0.84 | *IFFI* ^b^ | 1.34 | *INNI* ^c^ | 4.72 |  |  |
| *INCN* ^e^ | 0.32 | *FFFF* ^a^ | 0.60 | *IFNF* ^a^ | 0.85 | *FFNI* ^b^ | 1.35 | *NFNN* ^c^ | 4.72 |  |  |
| *ICCN* ^e^ | 0.33 | *INFN* ^b^ | 0.61 | *NCCI* ^d^ | 0.86 | *IFNI* ^b^ | 1.36 | *CNNI* ^d^ | 4.85 |  |  |
| *INCN* ^b^ | 0.33 | *NNCI* ^e^ | 0.61 | *NNNI* ^d^ | 0.87 | *NCNI* ^b^ | 1.38 | *CNIC* ^d^ | 5.42 |  |  |
| *ICCN* ^b^ | 0.33 | *IFNI* ^a^ | 0.61 | *NNCI* ^a^ | 0.87 | *CCNI* ^e^ | 1.43 | *INNI* ^b^ | 5.91 |  |  |
| *IFFF* ^c^ | 0.34 | *NNCI* ^d^ | 0.62 | *ICNI* ^a^ | 0.87 | *NCNI* ^d^ | 1.45 | *INNF* ^c^ | 5.97 |  |  |
| *FNFF* ^a^ | 0.35 | *NNFF* ^a^ | 0.63 | *ICNI* ^a^ | 0.87 | *NFNI* ^b^ | 1.49 | *INNN* ^b^ | 6.67 |  |  |
| *NFFN* ^b^ | 0.35 | *NNIN* ^c^ | 0.63 | *NCNI* ^a^ | 0.88 | *NNII* ^b^ | 1.50 | *NCCI* ^b^ | 6.74 |  |  |
| *INCI* ^b^ | 0.35 | *ICCN* ^d^ | 0.63 | *CCNN* ^a^ | 0.89 | *INNF* ^a^ | 1.53 | *NCIC* ^d^ | 6.82 |  |  |
| *FFNF* ^b^ | 0.35 | *ICCN* ^d^ | 0.63 | *FNNI* ^a^ | 0.89 | *CCNI* ^e^ | 1.55 | *INNN* ^e^ | 6.92 |  |  |
| *FFNN* ^b^ | 0.35 | *NFNI* ^c^ | 0.64 | *NCNI* ^d^ | 0.89 | *NCNI* ^e^ | 1.61 | *NCCI* ^a^ | 8.30 |  |  |
| *FFFF* ^b^ | 0.36 | *ININ* ^c^ | 0.65 | *NCCN* ^a^ | 0.89 | *CCNI* ^b^ | 1.62 | *IINN* ^b^ | 8.82 |  |  |
| *NFNF* ^b^ | 0.36 | *FFFN* ^c^ | 0.65 | *IFNN* ^a^ | 0.89 | *NCNI* ^b^ | 1.74 | *NCCI* ^b^ | 9.56 |  |  |
| *ICNN* ^b^ | 0.36 | *FNNN* ^a^ | 0.66 | *IINN* ^c^ | 0.90 | *ICNI* | 1.80 | *ICCI* ^b^ | 9.56 |  |  |
| *FFNN* ^a^ | 0.36 | *INFF* ^b^ | 0.67 | *NIIN* ^a^ | 0.91 | *NCNI* ^a^ | 1.82 | *INFI* ^b^ | 9.75 |  |  |
| *NFNF* ^a^ | 0.36 | *FFNF* ^a^ | 0.68 | *IINN* ^a^ | 0.92 | *INNI* ^a^ | 1.88 | *IICC* ^d^ | 11.46 |  |  |
| *FNFF* ^c^ | 0.37 | *IFNF* ^b^ | 0.68 | *CCNI* ^d^ | 0.93 | *NNFI* ^b^ | 1.92 | *CNNI* ^a^ | 11.52 |  |  |
| *ICNN* ^e^ | 0.37 | *ICCN* ^e^ | 0.69 | *ICNN* ^a^ | 0.95 | *INNN* ^a^ | 1.95 | *ICCI* ^a^ | 16.35 |  |  |
| *INFF* ^c^ | 0.38 | *NNII* ^c^ | 0.70 | *ININ* ^a^ | 0.95 | *NCCI* ^d^ | 2.01 | *INFI* ^c^ | 16.92 |  |  |
| *NNFN* ^b^ | 0.38 | *NFNI* ^a^ | 0.70 | *NNII* ^a^ | 0.95 | *NFFI* ^b^ | 2.05 | *NICC* ^d^ | 20.20 |  |  |
| *NFFF* ^b^ | 0.38 | *IIII* ^b^ | 0.70 | *INCN* ^a^ | 0.97 | *ICNI* ^b^ | 2.11 | *NCCI* ^a^ | 21.20 |  |  |
| *NFNF* ^c^ | 0.38 | *FNNF* ^a^ | 0.72 | *NNNN* ^a^ | 1 | *INNF* ^b^ | 2.14 | *INFI* ^a^ | 23.29 |  |  |
| *NNFF* ^b^ | 0.39 | *ICNN* ^d^ | 0.73 | *NNNN* ^b^ | 1.00 | *INNN* ^c^ | 2.25 | *IFFI* ^a^ | 24.61 |  |  |
| *ININ* ^b^ | 0.39 | *INCN* ^d^ | 0.73 | *NNNN* ^c^ | 1.00 | *NNNI* ^c^ | 2.32 | *IICC* ^e^ | 24.64 |  |  |
| *FFNI* ^c^ | 0.39 | *CCNI* ^a^ | 0.73 | *IFNI* ^c^ | 1.01 | *FNNI* ^b^ | 2.35 | *NICC* ^e^ | 27.61 |  |  |
| *NNCI* ^b^ | 0.40 | *INCI* ^d^ | 0.74 | *NNIN* ^a^ | 1.02 | *ICNI* ^b^ | 2.37 | *CNIC* ^e^ | 28.84 |  |  |
| *FFFN* ^b^ | 0.41 | *CNCN* ^a^ | 0.74 | *INCI* ^a^ | 1.07 | *CNNI* ^e^ | 2.37 | *CINC* ^d^ | 31.19 |  |  |
| *NIIN* ^b^ | 0.42 | *NNCI* ^d^ | 0.75 | *CNNN* ^a^ | 1.08 | *NNNI* ^b^ | 2.60 | *NCIC* ^e^ | 36.53 |  |  |
| *NNCI* ^b^ | 0.42 | *FNNF* ^b^ | 0.76 | *NNNF* ^c^ | 1.08 | *FFNF* ^c^ | 2.62 | *CINC* ^e^ | 46.65 |  |  |
| *NNIN* ^b^ | 0.43 | *INFN* ^a^ | 0.77 | *ICCI* ^d^ | 1.08 | *IIII* ^c^ | 2.71 | *NNCC* ^a^ | 47.26 |  |  |
| *ICCN* ^b^ | 0.44 | *INFF* ^a^ | 0.77 | *NINI* ^a^ | 1.09 | *ICCI* ^e^ | 2.72 | *CNIC* ^a^ | 49.31 |  |  |
| *NNNF* ^b^ | 0.45 | *IFFF* ^b^ | 0.78 | *INNI* ^d^ | 1.11 | *CNNI* ^b^ | 2.82 | *NCNC* ^a^ | 50.95 |  |  |
| *NFFN* ^c^ | 0.45 | *CCNI* ^a^ | 0.79 | *NINN* ^a^ | 1.12 | *NINN* ^c^ | 3.04 | *NNNC* ^a^ | 54.42 |  |  |
| *NFFF* ^a^ | 0.48 | *NCNN* ^a^ | 0.79 | *NNNI* ^a^ | 1.13 | *FNNF*^c^ | 3.19 | *CNNC* ^a^ | 56.86 |  |  |

**^a^** Promoter activity characterized in wild type E. coli (black)

**^b^** Promoter activity characterized in Δihf *E. coli* (blue)

**^c^** Promoter activity characterized in Δfis *E. coli* (red)

**^d^** Promoter activity characterized wild type *E. coli* in presence of glucose (yellow)

**^e^** Promoter activity characterized in Δihf *E. coli* in presence of glucose (green)
